# Bilateral multifocal nephrogenic adenomas arising in the renal pelves: a case report and review of the literature

**DOI:** 10.3389/fonc.2026.1866150

**Published:** 2026-07-09

**Authors:** Shuting Li, Zhenyong Wu, Xiaodong Lin, Lifang Ye, Wenqing Xu, Zeyun Lin, Xin Zeng

**Affiliations:** 1 Guangzhou Development District Hospital, Guangzhou, China; 2 First Affiliated Hospital of Guangzhou Medical University, Guangzhou, China

**Keywords:** atypical, bilateral, case report, multifocal, nephrogenic adenoma, renal pelvis

## Abstract

**Background:**

Nephrogenic adenoma (NA) is a benign epithelial lesion of the genitourinary tract resulting from the reimplantation and proliferation of exfoliated renal tubular cells at sites of urothelial injury. It most commonly arises in the bladder, while involvement of the renal pelvis is exceedingly rare. Bilateral multifocal presentation with atypical histologic features has not been previously reported.

**Case presentation:**

A 50-year-old male with a history of bladder stone lithotripsy presented with painless gross hematuria and lumbodorsal aching of nine months’ duration. Preoperative workup included three urine cytology examinations, which showed atypical cells suspicious for malignancy, and an outside hospital biopsy of the left renal pelvis. Review of the biopsy at our institution revealed no definitive malignant components (the specimen contained renal parenchyma, fibromuscular tissue, and scant urothelium with chronic inflammation), rendering the diagnosis indeterminate. Ultrasonography revealed bilateral multiple renal calculi and multiple solid masses in the bilateral renal collecting systems, suggestive of bilateral renal pelvic carcinoma. Given the bilateral disease and diagnostic uncertainty, the patient underwent staged percutaneous nephroscopic tumor resections. The right-sided lesion showed NA with focal atypia (CK7+, PAX8+, P504S+, CK20−, GATA-3−, Ki-67 ~10%). Two months later, the patient was readmitted for left-sided lesions. Non-contrast computed tomography (CT) demonstrated multiple nodular and strip-like lesions at the left renal pelvis and ureteropelvic junction, with the largest measuring 14 × 8 mm, accompanied by mild dilatation of the left pelvicalyceal system. Left percutaneous nephroscopic tumor resection was performed, and pathology confirmed atypical NA (PAX8+, EMA+, TFE3+, focal CK7/CK20/P504S/CAIX+, Ki−67 ~10%, p53 patchy ~80%). Specimens were fragmented, precluding margin assessment, though no macroscopic residual tumor was observed intraoperatively. Follow-up at 10 months showed no recurrence or metastasis.

**Conclusion:**

This is the first report of bilateral multifocal NA arising in the renal pelvis with atypical histologic features and an elevated Ki-67 proliferation index. This case expands the known morphological spectrum of NA and highlights important diagnostic challenges. Urologists and pathologists should be aware of this rare entity to avoid misdiagnosis as bilateral renal pelvic carcinoma and subsequent overtreatment.

## Introduction

Nephrogenic adenoma (NA), also termed nephrogenic metaplasia, is a rare benign proliferative lesion that may involve any site along the urinary tract. NA was initially reported by Davis in 1949, who characterized it as a “hamartoma of the bladder” based on its histopathological appearance. In 1950, Friedman and Kuhlenbeck described eight additional cases and introduced the term “nephrogenic adenoma, ” noting the morphological similarity of the lesion to mesonephric tubules (1). This nomenclature has since been widely adopted. It demonstrates a male predominance and most commonly arises in the bladder (80%), followed by the urethra (15%) and ureter (5%), while involvement of the renal pelvis is exceedingly rare (2). Clinical manifestations are site-dependent and typically encompass painless hematuria and irritative voiding symptoms, including frequency, urgency, and dysuria. The pathogenesis of NA remains poorly defined. Venyo (3) postulated that repeated urothelial irritation resulting from recurrent inflammation, urolithiasis, prior urological surgery, or mechanical injury may represent key contributing factors in the etiology of NA. It has also been reported to occur subsequent to renal transplantation or intravesical Bacillus Calmette-Guerin (BCG) therapy (4).

The diagnosis of bilateral NA arising in the renal pelvis is particularly challenging, as this exceptionally rare entity may mimic other conditions such as carcinoma, often leading to preoperative misdiagnosis. To the best of our knowledge, this is the first reported case of multifocal NAs localized to the renal pelvis in the English literature. Herein, we highlight its clinicopathological features, diagnostic pitfalls, and management to raise awareness of this rare benign entity among clinicians and pathologists. We present the following case in accordance with the CARE guideline.

## Case presentation

A 50-year-old male presented to our institution in March –2025 with spontaneous, painless gross hematuria without an identifiable precipitating cause, which was associated with a dull aching sensation in the lumbodorsal region. His medical history was significant for a bladder stone lithotripsy performed at an outside hospital more than ten years prior. There was no family history of urinary tract malignancies, renal diseases, or similar lesions in any first-degree relatives. Preoperative evaluation included three urine cytology examinations, which showed atypical cells suspicious for malignancy, and an outside hospital biopsy of the left renal pelvis. Review of this biopsy at our institution revealed no definitive malignant components, rendering the diagnosis indeterminate.

**Figure 1:**
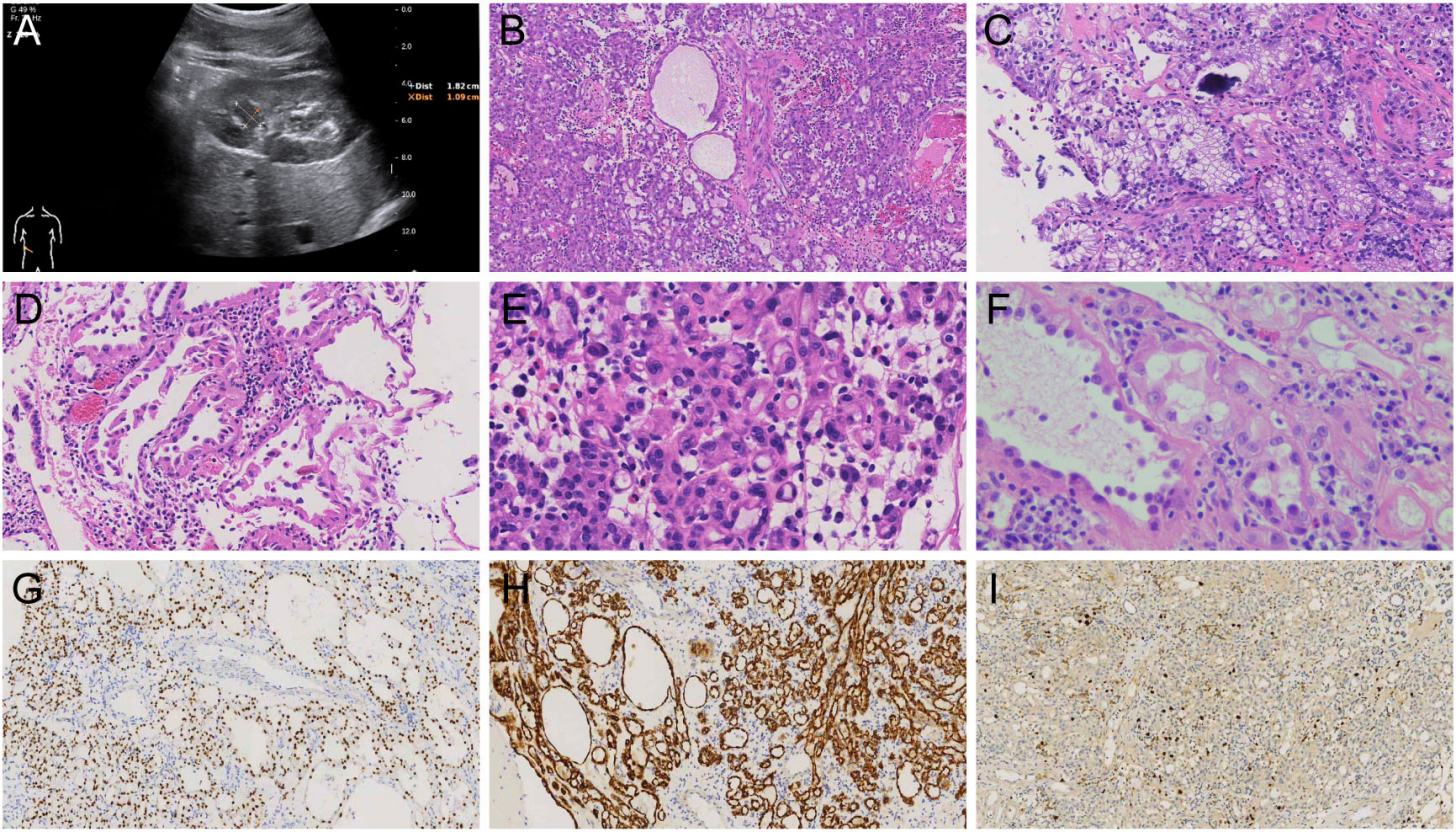
Imaging and pathological findings of the right renal lesion. **(A)** Abdominal ultrasonography showing a solid mass in the right renal collecting system. **(B)** Histopathological examination demonstrating microcystic and tubular architecture (HE, ×100). **(C)** Clear cell change with focal calcification (HE, ×200). **(D)** Oncocytic cells (HE, ×200). **(E)** Focal signet-ring-like cells with occasional atypical cells (HE, ×400). **(F)** Hobnail cells with focal cytologic atypia and visible nucleoli (HE, ×400). **(G)** Immunohistochemistry showing diffuse nuclear positivity for PAX8 (×100). **(H)** Immunohistochemistry showing positivity for P504S (AMACR) (×100). **(I)** Ki-67 proliferation index was approximately 10% (×100).

Abdominal ultrasonography (**Figure 1A**) revealed bilateral multiple renal calculi and multiple solid masses within the bilateral renal collecting systems, suggestive of bilateral renal pelvic carcinoma, accompanied by upper calyceal effusion and hematoma in the right kidney.

The patient underwent right percutaneous nephroscopic tumor resection under general anesthesia for the right renal pelvic mass. Intraoperative frozen section was not performed. Postoperative histopathological examination of the right-sided lesion revealed NA with focal atypia. Histopathological examination of the right-sided lesion showed microcystic and tubular architecture (**Figure 1B**), clear cell change with focal calcification (**Figure 1C**), and the presence of oncocytic cells (**Figure 1D**). Focal signet-ring-like cells with occasional atypical cells (**Figure 1E**) and hobnail cells with focal cytologic atypia and visible nucleoli (**Figure 1F**) were also observed. Immunohistochemically, the lesional cells were positive for CK7 (majority+), PAX-8 (**Figure 1G**), P504S (AMACR) (**Figure 1H**), and CK19, while negative for CK20, GATA-3, PSA, and CK5/6. The Ki-67 proliferation index was approximately 10% (**Figure 1I**). The patient was discharged after an uneventful recovery.

**Figure 2:**
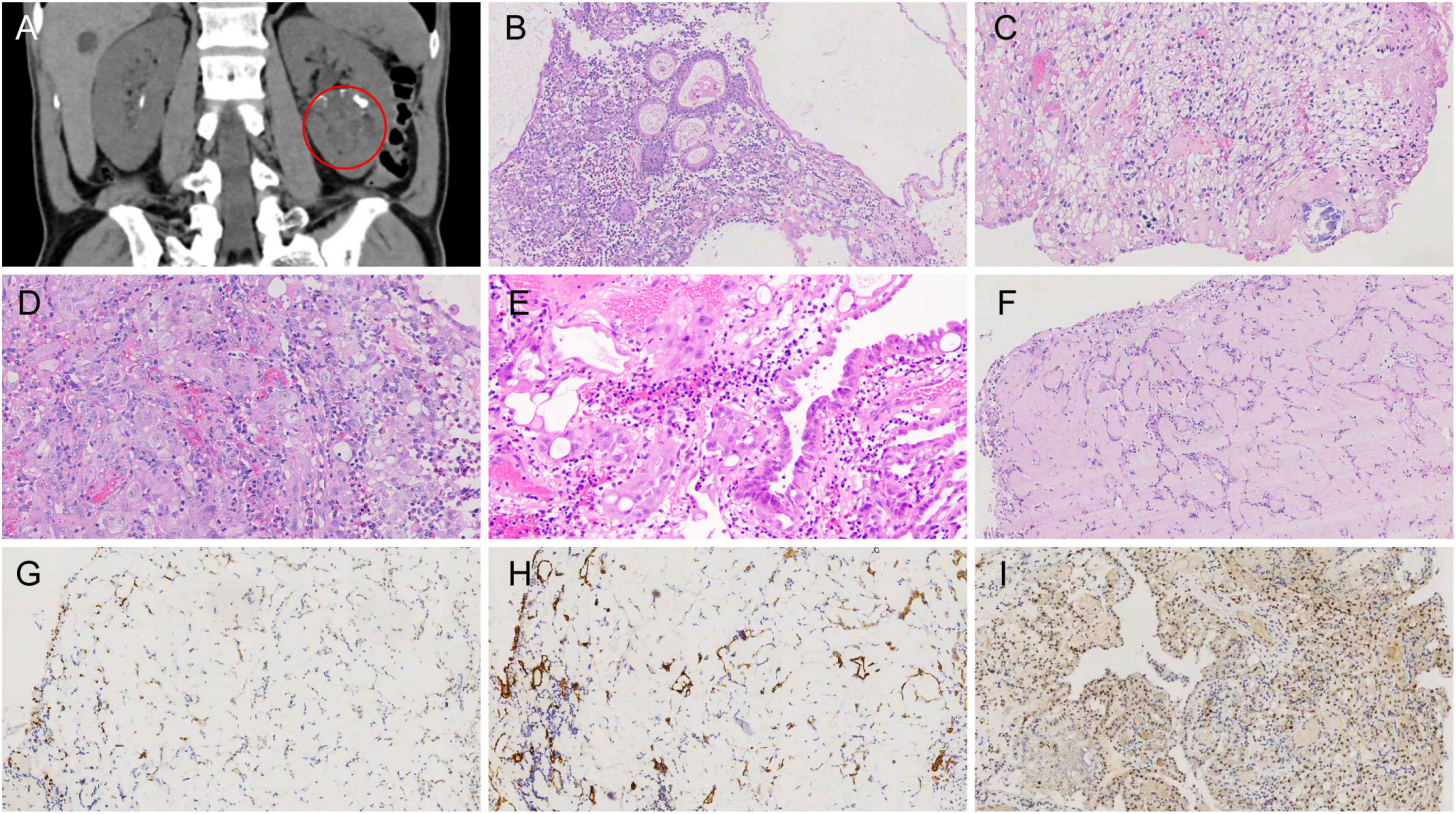
Imaging and pathological findings of the left renal lesion. **(A)** Non-contrast CT showing multiple nodular and strip-like hyperdense lesions at the left renal pelvis (red circle). **(B)** Histopathological examination demonstrating focal squamous metaplasia (HE, ×100). **(C)** Sheets of clear cells with calcification, not meeting the diagnostic criteria for clear cell renal cell carcinoma (HE, ×200). **(D)** Focal atypical cells (HE, ×200). **(E)** Atypical cells with hobnail morphology (HE, ×400). **(F)** Focal fibromyxoid change (HE, ×100). **(G)** Immunohistochemistry showing diffuse nuclear positivity for PAX8 (×100). **(H)** Immunohistochemistry showing focal positivity for P504S (AMACR) (×100). **(I)** p53 immunohistochemistry demonstrated diffuse positivity in approximately 80% of cells (patchy pattern), indicating aberrant p53 expression (×100).

Two months later, the patient was readmitted for evaluation of the left-sided lesions. Non-contrast CT (**Figure 2A**) of the abdomen revealed multiple calculi in the left kidney, accompanied by mild dilatation and fluid accumulation in the adjacent upper left ureter and the left renal pelvicalyceal system. A mixed slightly hyperdense lesion was observed in the left renal pelvis. Additionally, multiple nodular and strip-like hyperdense and mixed slightly hyperdense lesions were identified at the left renal pelvis and ureteropelvic junction. Given the bilateral disease and continued diagnostic uncertainty, the patient subsequently underwent left percutaneous nephroscopic tumor resection for the left renal pelvic masses under general anesthesia. Intraoperative frozen section was not performed during the left-sided procedure either. Histopathological examination of the left-sided lesions confirmed atypical NA, demonstrating the typical features of NA characterized by microcystic and tubular structures lined by a single layer of cuboidal epithelium. In addition to these classic findings, the lesion exhibited several unusual morphological alterations, including focal squamous metaplasia (**Figure 2B**), sheets of clear cells with focal calcification that fell short of the diagnostic criteria for clear cell renal cell carcinoma (**Figure 2C**), focal atypical cells (**Figure 2D**), atypical cells with hobnail morphology (**Figure 2E**), and focal fibromyxoid change (**Figure 2F**). Immunohistochemistry showed positivity for PAX-8 (**Figure 2G**), pan-cytokeratin (CK), EMA, TFE3, and focal positivity for CK7, CK20, P504S (AMACR) (**Figure 2H**), Vimentin, CD10, and CAIX (small focus). P53 demonstrated a patchy staining pattern in approximately 80% of cells (**Figure 2I**), a pattern consistent with a wild−type (reactive) phenotype, and the Ki-67 proliferation index was approximately 10% in hot spots. The resected specimens were fragmented, therefore, margin assessment could not be performed. However, no macroscopic residual tumor was observed intraoperatively.

The patient was followed up for 10 months postoperatively. Clinical examination and repeat abdominal imaging at the 10-month follow-up showed no evidence of local recurrence or distant metastasis. A timeline summarizing the key clinical events is presented in **Table 1**.

**Table 1:** Timeline of clinical events.

>10 years prior	Bladder stone lithotripsy at outside hospital
April 2024	Presented with painless gross hematuria and lumbodorsal aching
March 2025	Outside hospital: contrast−enhanced CT + left renal biopsy (xanthogranulomatous pyelonephritis? malignancy not excluded)
March 2025	Admitted to our institution; three urine cytology exams (atypical cells, suspicious); biopsy review (no definitive malignancy); ultrasound: bilateral renal calculi and bilateral renal pelvic masses
March 2025	Right percutaneous nephroscopic tumor resection; pathology: NA with focal atypia (Ki-67 about 10%+)
May 2025	Readmission; CT: left renal pelvic and ureteropelvic junction lesions
May 2025	Left percutaneous nephroscopic tumor resection; pathology: atypical NA (Ki-67 about 10%+)
March 2026	10-month follow-up: no recurrence or metastasis; patient lost to follow-up thereafter

## Literature review

A comprehensive review of the English and Chinese literature was performed using the PubMed and CNKI databases with the keywords “nephrogenic adenoma”, “nephrogenic metaplasia”, and “renal pelvis”. To date, a limited number of cases of NA originating in the renal pelvis have been reported. A 2021 review by Zhang et al. (5) identified 19 previously reported cases of renal pelvic NA and additionally reported one new case of their own, bringing the total to 20 cases up to that time. It should be noted that demographic information was incomplete for a subset of the cases in the review. In 2022, Li et al. (6) reported a series of 43 fibromyxoid NAs, of which 7 cases (16.3%) occurred in the kidney and 4 cases involved the renal pelvis. In 2023, Ren et al. (7) reported a pure fibromyxoid variant of NA arising in the renal pelvis in a 37-year-old female, the first such description in this location. In 2024, Wang et al. (8) reported a clinicopathological analysis of 13 NA cases, of which 2 cases involved the renal pelvis. A summary of the key reported cases is presented in **Table 2**.

**Table 2:** Summary of reported cases of NA arising in the renal pelvis.

Author (Year)	Cases	Age/sex	Laterality	Multifocality	Atypia
Zhang et al. (2021) (5)- literature review	19*	Range: 20-73y Male: Female=3:4	All unilateral	All unifocal	Not reported
Zhang et al. (2021) (5) - own case	1	46y/Male	Unilateral	Unifocal	Yes
Li et al. (2022) (6)	4	92y/Female	Unilateral	Unifocal	Not reported
		63y/Male	Unilateral	Unifocal	Not reported
		25y/Female	Unilateral	Unifocal	Not reported
		26y/Female	Unilateral	Unifocal	Not reported
Ren et al. (2023) (7)	1	37y/Female	Unilateral	Unifocal	No
Wang et al. (2024) (8)	2	26y/Male	Unilateral	Unifocal	No
		41y/Female	Unilateral	Unifocal	No
Present case	1	50y/Male	Bilateral	Multifocal	Yes
Total	28				

*Among the 19 cases summarized by Zhang et al. (2021), detailed age and sex information was not reported for 12 cases.

As demonstrated in **Table 2**, all previously reported cases were unilateral and unifocal. Our case is the first to describe bilateral and multifocal involvement, addressing a significant gap in the literature.

## Discussion

NA is a rare benign metaplastic lesion of the urinary tract, most commonly arising in the bladder. Primary involvement of the renal pelvis is exceptionally rare, and bilateral multifocal presentation with atypical histologic features has not been previously reported. In this report, we present the first case of bilateral multifocal NA arising in the renal pelvis with atypical pathological features, including focal cytologic atypia and an elevated Ki-67 proliferation index (approximately 10%).

NA has been reported across a broad age range and shows a male predominance, with a male-to-female ratio of approximately 2:1 (5). The present case, a 50-year-old male, is consistent with this typical demographic profile. The most common presenting symptoms of NA are painless gross hematuria and flank or lumbodorsal discomfort, which are indistinguishable from those of renal pelvic malignancy. Our patient presented with both symptoms, consistent with prior reports. Of note, the patient had a remote history of bladder stone lithotripsy performed more than ten years prior to presentation. This antecedent urological procedure supports the proposed association between prior urothelial injury and the subsequent development of NA.

Critically, all previously reported cases of renal pelvic NA have been unilateral and unifocal. To the best of our knowledge, the present case is the first to describe bilateral and multifocal involvement of the renal pelvis, representing a unique presentation that expands the known anatomical spectrum of this entity.

The radiological presentation of NA is non-specific, overlapping with cystitis glandularis, papilloma, and urothelial carcinoma, which poses a diagnostic challenge before surgery. Reflecting this difficulty, the present case was presumed malignant on imaging, leading to surgical exploration. This case demonstrates that bilateral multifocal NA can be radiologically indistinguishable from renal pelvic carcinoma.

Preoperative workup included three urine cytology examinations, all showing atypical cells suspicious for malignancy. The patient also underwent a left renal pelvis biopsy at an outside hospital. Our institution reviewed the biopsy but found no definitive malignant components. The diagnosis thus remained indeterminate. Together with the bilateral nature of the lesions, these findings created a diagnostic dilemma that warranted surgical intervention for definitive tissue diagnosis.

The pathogenesis of NA is thought to involve the reimplantation and proliferation of exfoliated renal tubular cells at sites of urothelial injury, a theory first proposed by Friedman and Kuhlenbeck in 1950 (1). Recognized risk factors include prior urological surgery, urolithiasis, recurrent urinary tract infections, chronic inflammation, intravesical BCG therapy, renal transplantation, and long-term dialysis (9). In the present case, the patient’s history of bladder stone lithotripsy more than ten years prior may have served as the initial insult. This procedure likely caused chronic, subclinical inflammation of the urothelium, creating a favorable environment for the implantation and proliferation of shed renal tubular cells. The bilateral and multifocal nature of the lesions in this case is particularly noteworthy. Unlike all previously reported unilateral, unifocal cases, the involvement of both renal pelves and the presence of multiple lesions suggest a widespread process. This is likely a result of the patient’s long-standing history of bilateral renal calculi, which would have caused chronic, widespread inflammation of the urothelium in both kidneys. In this context of persistent injury, multiple independent foci of NA may arise, a phenomenon not previously described in the literature.

Microscopically, conventional NA is characterized by small tubules and microcysts within the lamina propria, surface papillary projections, and a single layer of flat, cuboidal, or low columnar epithelial cells with uniform, bland nuclei. Mitotic figures are typically rare or absent. The present case exhibited these characteristic architectural features in both renal pelvic lesions. However, several atypical features were observed. The right-sided lesion was diagnosed as “NA with focal atypia, ” while the left-sided lesion was diagnosed as “atypical NA”.

In addition to these atypical features, the left-sided lesion exhibited several other unusual morphological alterations, including focal squamous metaplasia, sheets of clear cells with calcification, and focal fibromyxoid change. The clear cell change was notable but did not meet the diagnostic threshold for clear cell renal cell carcinoma (RCC). Fibromyxoid change is a rare morphological variant of NA that can mimic mesenchymal tumors or myxoid malignancies, particularly on small biopsy specimens (6, 7, 10, 11). The presence of this finding in our case further expands the known morphological spectrum of renal pelvic NA.

Immunohistochemistry (IHC) is indispensable for confirming the diagnosis of NA and, more critically, for excluding histological mimics. In the present case, IHC was essential in navigating several diagnostic pitfalls.

The lesional cells from both kidneys demonstrated strong positivity for PAX-8, confirming a nephrogenic lineage and effectively ruling out a primary urothelial origin. This was further supported by the absence of GATA-3 and lack of diffuse CK20 expression, both of which argue against urothelial carcinoma. Both lesions showed positivity for P504S (AMACR), a marker typically associated with papillary renal cell carcinoma (pRCC). This finding represents a well-recognized diagnostic pitfall in NA. However, the diagnosis of pRCC was excluded by the lack of destructive growth, bland cytology, and the overall histologic architecture consistent with NA. The left-sided lesion exhibited focal expression of CAIX and TFE3, along with a patchy p53 staining pattern in approximately 80% of cells. These findings did not meet the diagnostic thresholds for clear cell RCC, which would require diffuse, strong CAIX expression, nor for TFE3-rearranged RCC, which would show diffuse, strong TFE3 expression. The patchy staining pattern in the left−sided lesion is consistent with a wild−type (reactive) phenotype rather than a mutant−type expression, as true aberrant p53 expression typically shows an “all−or−nothing” profile (diffuse strong positivity or complete negativity). This pattern likely reflects underlying chronic inflammatory stress rather than true malignant transformation.

The most notable aspect of this case is the presence of atypical histologic features and an elevated Ki-67 proliferation index. Typical NA is characterized by a low Ki-67 index, typically less than 5% (12). In contrast, our patient’s lesions exhibited a Ki-67 proliferation index of approximately 10% in both renal pelves (10% in the right kidney and approximately 10% in hot spots in the left kidney). This finding is unusual for conventional NA and raises the critical differential diagnosis of low-grade malignancies, particularly papillary renal cell carcinoma, collecting duct carcinoma, or low-grade urothelial carcinoma. Although it was traditionally believed that NA follows a benign course without malignant transformation even in the presence of significant cytologic atypia, subsequent reports have documented malignant transformation (9, 13). Nevertheless, in the present case, several findings support the benign nature of the lesion despite its atypical features: the overall architecture was characteristic of NA with no destructive growth, the lesional cells retained PAX-8/PAX-2 expression confirming a nephrogenic lineage, no significant mitotic activity or invasion was observed despite the elevated Ki-67 index, and the patient remained free of recurrence or metastasis at 10-month follow-up. We therefore interpret these atypical features as an unusual morphological variant of NA rather than evidence of malignancy. This case expands the known histological spectrum of NA and highlights the importance of careful correlation of morphological, immunohistochemical, and clinical findings to avoid misdiagnosis.

There is currently no standardized treatment protocol for NA. Minimally invasive endoscopic management is commonly employed (5); however, endoscopic biopsy often fails to achieve complete resection, which may be associated with a risk of local recurrence. Surgical excision represents another widely used approach. Notably, studies have reported that NA is prone to recurrence following surgical resection, particularly in pediatric patients, with recurrence rates ranging from 30% to 80% (14).

In the present case, given the bilateral disease and diagnostic uncertainty, a staged percutaneous nephroscopic tumor resection was performed. This approach was selected to obtain definitive tissue diagnosis while preserving renal function, which would have been lost with bilateral radical nephroureterectomy. The resected specimens were fragmented, precluding margin assessment; however, no macroscopic residual tumor was observed intraoperatively, and 10-month follow-up imaging confirmed no recurrence. This strategy aligns with the principle of nephron-sparing management for bilateral upper tract lesions, where preservation of renal function is prioritized alongside oncological control. A radical nephroureterectomy was discussed with the patient and kept as a backup plan in case the final pathology had confirmed high-grade malignancy.

The prognosis of NA following complete excision is generally favorable, with most cases following a benign course. Although NA is classically considered a metaplastic lesion, it can lead to serious consequences such as urinary tract obstruction and carries a potential risk of malignant transformation, with clear cell adenocarcinoma (CCA) being the most common type (15). Therefore, regular postoperative follow-up is warranted. In addition, frequent endoscopic examination and instrumentation should be avoided, as they may predispose to recurrence of NA. In the present case, the patient remained free of recurrence or metastasis at 10-month follow-up, consistent with the generally favorable outcome of NA. Nevertheless, given the potential for late recurrence or rare malignant transformation, long-term surveillance is recommended.

## Limitations

This study has several limitations. First, as a single case report, the findings may not be generalizable to other patients with NA. Second, the follow-up period was limited to 10 months; longer-term surveillance would be valuable to further confirm the benign course of atypical NA. Third, the resected specimens were fragmented, which precluded histopathological margin assessment. Fourth, the absence of molecular analysis limits our understanding of the biological basis of the atypical features observed, particularly the elevated Ki-67 index and aberrant p53 expression. Fifth, as discussed above, detailed demographic and clinical data were not available for 12 of the 28 previously reported cases in the literature, which may introduce reporting bias into our comparative analysis. Sixth, intraoperative frozen section was not performed. In urological practice, frozen section is generally reserved for specific margin evaluations rather than for diagnostic confirmation of renal masses, and its reliability in upper urinary tract lesions is limited. Nonetheless, we recognize this as a limitation of our approach.

## Conclusions

We report the first case of bilateral multifocal NA arising in the renal pelvis with atypical histologic features, including focal cytologic atypia and an elevated Ki-67 proliferation index (approximately 10%). This case expands the known anatomical and morphological spectrum of NA and highlights important diagnostic challenges. Urologists and pathologists should be aware of this rare entity to avoid misdiagnosis as bilateral renal pelvic carcinoma and subsequent overtreatment. In patients with a history of chronic urothelial injury, bilateral multifocal renal pelvic masses should include NA in the differential diagnosis. A staged, nephron-sparing surgical approach with careful postoperative surveillance is a reasonable strategy in such settings, particularly when preoperative diagnostic workup is inconclusive.

## Data Availability

The original contributions presented in the study are included in the article/supplementary material. Further inquiries can be directed to the corresponding author/s.
